# Baseline quantitative HBcAb strongly predicts undetectable HBV DNA and RNA in chronic hepatitis B patients treated with entecavir for 10 years

**DOI:** 10.1038/s41598-021-92757-0

**Published:** 2021-06-28

**Authors:** Xi Zhang, Xiaocui An, Lei Shi, Xueliang Yang, Yunru Chen, Xiaojing Liu, Jianzhou Li, Feng Ye, Shumei Lin

**Affiliations:** 1 Department of Infectious Diseases, First Affiliated Hospital of Xi’an Jiaotong University, No. 277 Yanta West Road, Xi’an City, 710061 Shaanxi Province China; 2 Department of Infectious Diseases, Hospital of Traditional Chinese Medicine of Yuyang District, Yulin, 719000 China

**Keywords:** Gastroenterology, Hepatology, Infectious diseases, Hepatitis

## Abstract

The predictive effect of quantitative anti-hepatitis B core on double-negative HBV DNA and RNA remains unstudied. We observed dynamic changes in this measure in chronic hepatitis B patients receiving entecavir for 10 years, evaluating its predictive value for double-negative HBV DNA and RNA. Twenty-seven chronic hepatitis B patients treated with entecavir for 10 years were enrolled in this study. Liver function, quantitative anti-hepatitis B core, hepatitis B surface and e antigens, HBV DNA and RNA were measured at baseline and each follow-up. Virological response was defined as double-negative HBV DNA and RNA; serological response was defined as hepatitis B e antigen seroconversion. After antiviral therapy, quantitative anti-hepatitis B core showed an overall downward trend. Patients with virological response had significantly higher quantitative anti-hepatitis B core levels than those without virological response at baseline. Patients with serological response also had higher quantitative anti-hepatitis B core levels than those without serological response at baseline and week 24. Baseline quantitative anti-hepatitis B core level was the only independent predictor for virological and serological responses. Baseline quantitative anti-hepatitis B core level was powerfully predictive of double-negative HBV DNA and RNA in chronic hepatitis B patients receiving long-term entecavir therapy.

## Introduction

Approximately 350 million people worldwide are infected with hepatitis B virus (HBV)^1^, which can lead to hepatitis, cirrhosis, hepatocellular carcinoma (HCC) and liver failure. Interferons (IFNs) and nucleotide analogue (NA) are the main anti-HBV drugs. For HBeAg-positive patients, virological response (VR) and serological response (SR) during therapy are defined as loss of serum HBV DNA and hepatitis B e antigen (HBeAg) seroconversion, respectively. Treatment discontinuation should not been considered until the patients have got alanine aminotransferase (ALT) normalization, VR and SR^2–5^. However, VR and SR don’t represent that HBV covalently closed circular DNA (cccDNA) has been cleared in hepatocytes. Patients with VR and SR still had high frequency of virological rebound and hepatitis relapse after discontinuation of NA.

Serum HBV RNA is an indicator of cccDNA activity in chronic hepatitis B (CHB) patients treated with NA^6,7^. Undetectable serum HBV RNA may indicate the transcriptional silencing of cccDNA^8,9^. Double-negative HBV DNA and RNA at end of NA treatment was considered as a potent marker for guiding discontinuation in HBeAg positive CHB patients by Fan et al.^10^. Accordingly, VR should be redefined as double-negative HBV DNA and RNA.

Hepatitis B core antibody (HBcAb) is an HBV-specific antibody that reflects the host immune response against HBV^11,12^. Yuan et al. first reported in 2013 that baseline quantitative anti-hepatitis B core (qAnti-HBc) levels may serve as a useful marker indicating an ongoing host immune activity against HBV^13^. Many studies have shown that baseline qAnti-HBc levels could serve as a useful marker for predicting SR in HBeAg-positive CHB patients during Peg-IFN and NA therapies^14–17^. In 2020, Fu et al. indicated that patients with baseline qAnti-HBc level ≥ 4.15log10 IU/mL and liver stiffness measurements ≥ 9.85 kPa had the highest rates of SR after 96 weeks of NA (entecavir, telbivudine or tenofovir disoproxil fumarate) therapy^18^. However, no studies have investigated the clinical value of qAnti-HBc levels for redefined VR (double-negative HBV DNA and RNA) following long-term NA therapy in CHB patients in real-life practice. Therefore, the aims of this study were to investigate dynamic changes of qAnti-HBc levels in CHB patients treated with entecavir for 10 years, and to evaluate its value in predicting redefined VR (double-negative HBV DNA and RNA).

## Results

### Demographic and clinical characteristics

Thirty-three CHB patients were enrolled in the study. In all, 27 patients with available serial samples were included in the analysis. The demographic, virological and clinical characteristics of the patients are summarized in Table 1. Patients were predominantly male (70.4%) with mean age of 32.41 ± 9.46 years, 77.8% were HBeAg positive, and 63% were genotype C. The means of baseline HBV DNA, HBV RNA, anti-HBc and ALT levels were 6.29 ± 1.21 log10 IU/mL, 5.39 ± 1.47 log10 copies/mL, 3.07 ± 0.87 log10 IU/mL and 104.73 ± 19.82 U/L, respectively.

**Table 1:** Demographics and baseline characteristics of entecavir-treated patients with chronic HBV infection.

Characteristics	N = 27
Male: no. (%)	19 (70.4%)
Age: mean(years)	32.41 ± 9.46
HBVfamily history no. (%)	20 (74.1%)
HBeAg positive no. (%)	21 (77.8%)
HBV genotype (B/C) (%)	37.0%/63.0%
Serum ALT(IU/L)	104.73 ± 19.82
Serum AST(IU/L)	81.55 ± 11.6
Serum HBV DNA (log10 IU/mL)	6.29 ± 1.21
Serum HBcAb (log10 IU/mL)	3.07 ± 0.87
Serum HBV RNA (log10 copies/mL)	5.39 ± 1.47

Continuous data are presented as means ± standard error, categorical data are shown as percentages.

### Therapy efficacy

**Figure 1:**
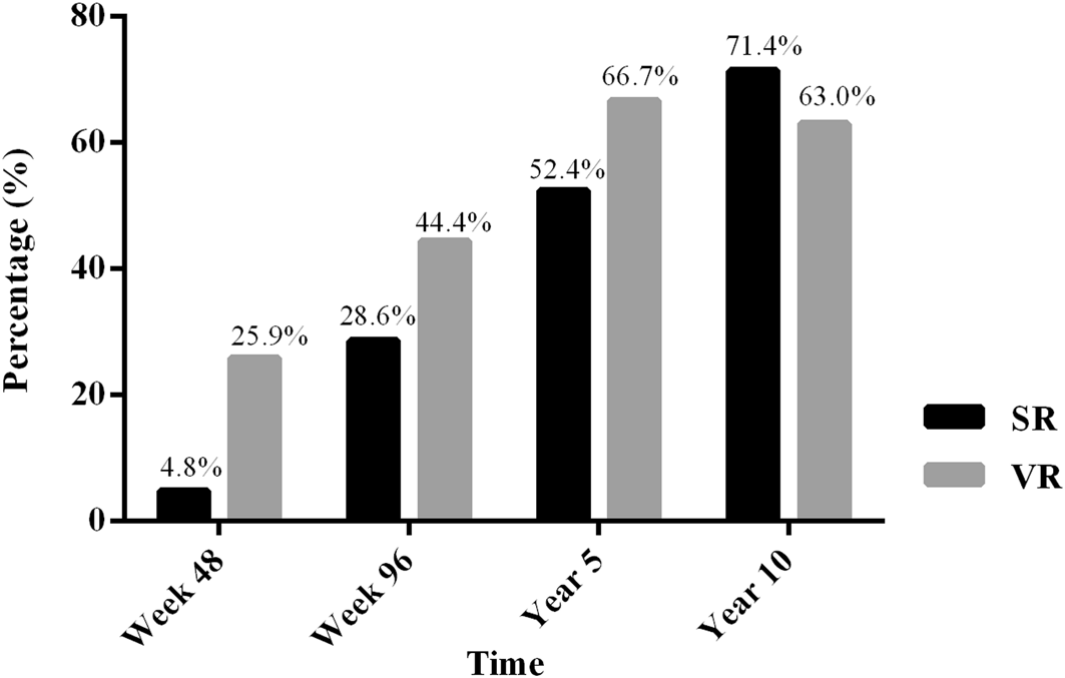
Percentage of patients achieving SR and VR during therapy (n = 27).

Of all 27 patients, 24 (88.9%) and one (3.7%) achieved ALT normalization and HBsAg loss, respectively, after 10 years of antiviral therapy. VR and SR during therapy increased from 25.9% and 4.8%, respectively, at week 48 to 63.0% and 71.4%, respectively, at year 10 (Fig. 1). The incidence of HCC, cirrhosis and death was 3.70% (1/27), 3.70% (1/27) and 0, respectively at year 10.

### Kinetics of qAnti-HBc, HBV DNA and HBV RNA during 10 years of antiviral therapy

**Figure 2:**
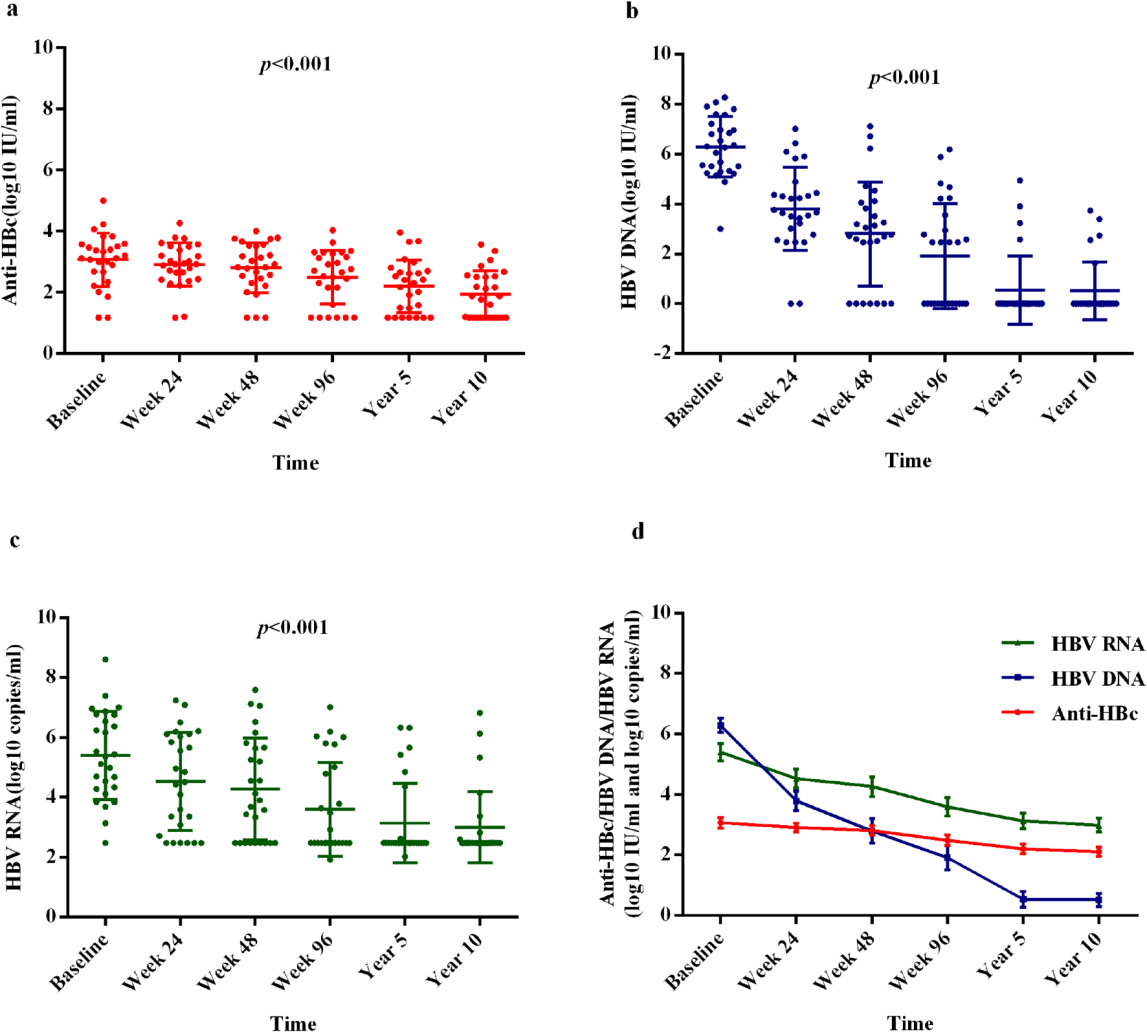
Changes of qAnti-HBc, HBV DNA and HBV RNA during 10 years’ entecavir therapy. (**a**) Scatter plot of qAnti-HBc. (**b**) Scatter plot of HBV DNA. (**c**) Scatter plot of HBV RNA. (**d**) Line chart of qAnti-HBc, HBV DNA and HBV RNA.

Serum qAnti-HBc, HBV DNA and HBV RNA were measured at baseline and after entecavir treatment at week 24, 48, and 96, and year 5 and 10 (Fig. 2a–c, Supplementary Table 1). Each parameter showed an overall significant downward trend with increasing duration of treatment (Fig. 2d, qAnti-HBc, p < 0.001; HBV DNA, p < 0.001; HBV RNA, p < 0.001).

### Kinetics of qAnti-HBc in patients with differing therapy responses

**Figure 3:**
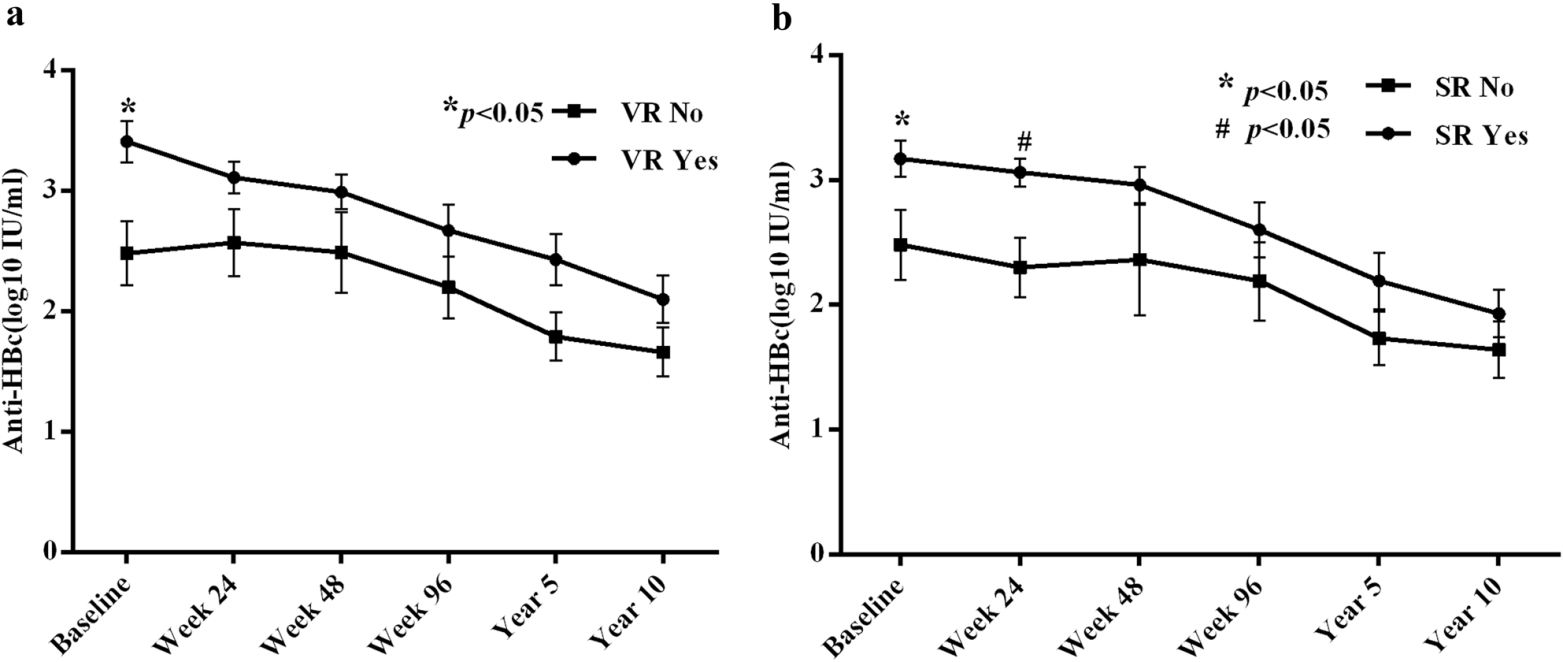
Kinetics of qAnti-HBc in patients stratified by (**a**) VR and (**b**) SR.

qAnti-HBc levels in patients stratified by treatment response were further analyzed (Fig. 3). Patients with VR had significantly higher baseline qAnti-HBc levels than those without VR (3.42 ± 0.71 log10 IU/mL vs 2.48 ± 0.84 log10 IU/mL, p = 0.005). In the HBeAg-positive cohort, patients with SR had significantly higher qAnti-HBc levels than those without SR at both baseline and week 24 (baseline, 3.17 ± 0.56 log10 IU/mL vs 2.24 ± 0.86 log10 IU/mL, p = 0.008; week 24, 3.05 ± 0.43 log10 IU/mL vs 2.29 ± 0.57 log10 IU/mL, p = 0.004).

### Correlation between baseline characteristics and therapy efficacy at year 10

To further evaluate baseline characteristics in predicting VR and SR, multivariate analyses were conducted with inclusion of age, HBV genotype and baseline levels of ALT, HBV DNA, HBV RNA and qAnti-HBc in the model. Regression analysis showed that baseline qAnti-HBc level was the only independent predictor for VR (odds ratio [OR] 0.091, 95% confidence interval [CI] 0.003–0.843, p = 0.038; Table 2) and SR (OR 0.018, 95% CI 0.001–0.479, p = 0.016; Table 3).

**Table 2:** Correlation between baseline characteristics and VR at year 10.

Variables	Univariate analysis			Multivariate analysis		
	OR	95%CI	p value	OR	95%CI	p value
Age	0.500	0.079–3.147	0.460	2.787	0.109–71.206	0.535
Baseline HBeAg state	0.267	0.026–2.699	0.263	0.579	0.029–11.709	0.721
HBV genotype	0.267	0.026–2.699	0.263	0.153	0.010–2.374	0.180
Baseline Anti-HBc (log10 IU/mL)	0.089	0.013–0.621	0.015	0.051	0.003–0.843	0.038
Baseline ALT (U/L)	0.387	0.092–2.521	0.194	0.600	0.025–14.316	0.752
Baseline HBV DNA (log10 IU/mL)	1.833	0.374–8.984	0.455	2.054	0.090–46.722	0.652
Baseline HBV RNA (log10 copies/mL)	4.278	0.798–22.928	0.090	5.845	0.427–79.980	0.186

**Table 3:** Correlation between baseline characteristics and SR at year 10.

Variables	Univariate analysis			Multivariate analysis		
	OR	95%CI	p value	OR	95%CI	p value
Age	0.727	0.094–5.633	0.760	2.995	0.091–98.928	0.539
HBV genotype	0.267	0.026–2.699	0.263	2.141	0.061–75.286	0.675
Baseline Anti-HBc (log10 IU/mL)	0.031	0.002–0.420	0.009	0.018	0.001–0.479	0.016
Baseline ALT (U/L)	0.438	0.061–3.160	0.413	1.457	0.029–73.552	0.851
Baseline HBV DNA (log10 IU/mL)	0.875	0.132–5.819	0.890	0.338	0.009–12.230	0.554
Baseline HBV RNA (log10 copies/mL)	1.750	0.242–12.642	0.579	1.903	0.048–75.396	0.732

### Performance of baseline qAnti-HBc level in predicting VR and SR

**Figure 4:**
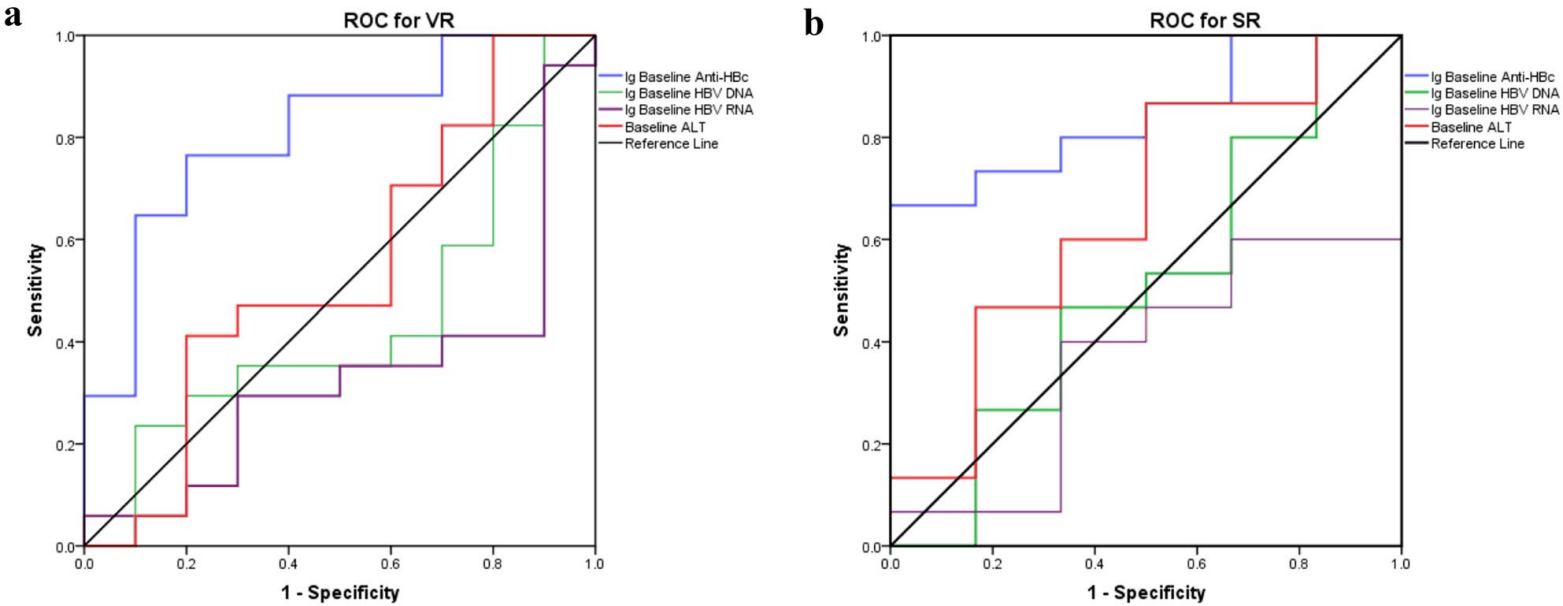
AUROC of baseline anti-HBc level in predicting (**a**) VR and (**b**) SR.

To evaluate the performance of baseline qAnti-HBc levels in predicting VR and SR, we examined the areas under the receiver operator characteristic curve (AUROC). As shown in Fig. 4, the AUROC of baseline qAnti-HBc was higher in predicting VR (0.812, p = 0.008) and SR (0.844, p = 0.016) at year 10 than baseline HBV DNA (VR, 0.441, p = 0.616; SR, 0.511, p = 0.938), HBV RNA (VR, 0.329, p = 0.145; SR, 0.367, p = 0.350) and ALT (VR, 0.541, p = 0.725; SR, 0.656, p = 0.276).

### Rates of VR and SR among patients with favorable baseline qAnti-HBc at year 10

The sum of sensitivity and specificity was maximal in predicting VR and SR at year 10 when the cut-off value was 3.1 log10 IU/mL. Patients were stratified into two groups according to the cut-off value. Eighty percent (8/10) and 100% (10/10) of patients with qAnti-HBc ≥ 3.1 log10 IU/mL achieved VR and SR, respectively, after 10 years of antiviral therapy. However, only 36.4% (4/11) and 45.5% (5/11) of patients in the group with qAnti-HBc < 3.1 log10 IU/mL achieved VR and SR, respectively, at year 10 (p = 0.006).

## Discussion

Baseline qAnti-HBc could predict HBeAg seroconversion in CHB patients treated with IFNs or NA^14–18^. The present study evaluated dynamic changes in qAnti-HBc in CHB patients during 10 years of entecavir therapy. We demonstrated that the mean qAnti-HBc level decreased gradually, and that baseline qAnti-HBc could serve as an independent predictor for HBeAg seroconversion. To our knowledge, this is the longest comprehensive and definitive analysis to assess the performance of qAnti-HBc levels in CHB patients treated with entecavir.

HBeAg seroconversion and HBV DNA suppression at the end of post-antiviral therapy follow-up are the two major endpoints associated with favorable outcomes in HBeAg-positive patients. However, HBeAg seroconversion and HBV DNA suppression are not equivalent to HBV cccDNA elimination in hepatic cells. HBV RNA directly derived from cccDNA can reflect the intrahepatic cccDNA level. Recently, the use of the redefined VR (double-negative HBV DNA and RNA) has been suggested to be a safe rule for cessation of NA therapy in CHB patients. However, no data have been reported regarding the predictive value of baseline qAnti-HBc levels for redefined VR in an NA-treated cohort. We were the first to discover baseline qAnti-HBc could serve as an independent predictor for the redefined VR. In addition, a baseline qAnti-HBc level of ≥ 3.1 log10 IU/mL was associated with higher rates of VR and SR in CHB patients treated with entecavir. However, the levels were lower than the results reported by previous studies^15,17,18^. Serum qAnti-HBc levels are closely related to host immune status and are strongly associated with hepatitis activity in CHB patients. Song et al.^11^ showed that the mean qAnti-HBc levels in patients in the immune clearance and HBeAg-negative hepatitis phases were significantly higher than those in patients in both the immune tolerance and low replicative phases. Serum qAnti-HBc levels were also positively correlated with ALT levels, inflammatory activity, significant fibrosis, HBV DNA, HBsAg and hepatitis B core-related antigen^19–21^. Compared with patients in previous studies, the patients in this study had lower levels of ALT and HBV DNA, and most of them were HBeAg-positive. These factors may account for the low baseline qAnti-HBc levels in the patients in this study.

Baseline HBV DNA, HBV RNA and ALT levels have been proven to be independently associated with HBeAg seroconversion in previous studies^15,22,23^. However, in the present study, when anti-HBc was included in the multivariate analysis in combination with either VR or SR, baseline HBV DNA, HBV RNA and ALT showed no correlation with either VR or SR. The AUROC values of HBV DNA, HBV RNA and ALT for VR and SR were also less than that of anti-HBc, indicating that anti-HBc levels had better predictive value than baseline HBV DNA, HBV RNA and ALT. HBcAb is produced by hepatitis B core antigen-activated B-cells, which could inhibit HBV replication through hepatocytotoxic effects and regulate the activity of CD4^+^ and CD8^+^ T cells by producing cytokines such as IFN-γ or IL-6^24,25^. Therefore, it is possible that a higher HBcAb level at baseline may reflect a better anti-viral response in CHB patients, which is associated with better prognosis after antiviral therapy. Baseline qAnti-HBc level may therefore be a potent biomarker for guiding NA discontinuation in CHB patients.

This study had several limitations. The major limitation was the relatively small sample size. Only 27 patients with CHB were included in this study, and therefore more patients are needed for future analyses. Furthermore, this was a single-center study; multi-center research should be conducted to explore in greater detail the clinical significance of qAnti-HBc in antiviral therapy. Additionally, we did not study the value of qAnti-HBc for the safe discontinuation of NA treatments.

In conclusion, our study showed that baseline serum qAnti-HBc was a powerful predictor of double-negative HBV DNA and RNA in CHB patients receiving long-term entecavir therapy.

## Methods

### Study population

CHB patients were given entecavir (0.5 mg/day, orally) after assigning informed consents and were followed between April 2007 and May 2018 at the department of infectious diseases of the First Affiliated Hospital of Xi’an Jiaotong University (Shaanxi, China). Serum samples of the patients were routinely collected and stored at -80℃. All patients were older than 16 years, with eGFR > 50 mL/(min × 1.73m^2^), had been positive for hepatitis B surface antigen (HBsAg) for longer than 6 months and had detectable serum HBV DNA. Reasons for exclusion were as follows: Compilated with A, C, D, E or other viral hepatitis; Compilated with acquired immunodeficiency syndrome; Decompensated liver cirrhosis (Child Pugh C); Taking other anti-HBV drug; Previous diagnosis of hepatocellular carcinoma; Compilated with autoimmune liver disease, alcoholic liver disease or cholestatic liver disease; With other serious medical conditions that affect follow-up compliance. The study was approved by the Ethics Committee of the First Affiliated Hospital of Xi’an Jiaotong University and was performed in accordance with relevant guidelines and regulations. Informed consent was obtained from the parents legally authorized representatives of subjects that were under 18.

### Clinical and laboratory evaluation

Routine demographic data collection (age, gender, family history of hepatitis B, etc.) was carried out for all the patients in the group. Liver function, blood routine, HBV DNA quantitative value, HBeAg quantitative value, HBsAg quantitative value and upper abdominal ultrasound were carried out at baseline, then liver function, blood routine, HBV DNA quantitative value, HBeAg quantitative/qualitative value, HBsAg quantitative/qualitative value and upper abdominal ultrasound were checked every 12 weeks at the first year, and 24 weeks from the second year to the end of study. HBV DNA was measured with COBAS TaqMan HBV test (ROCHE, USA, Lower limit of detection, 20 IU/mL). HBV genotype was determined by nested PCR using type-specific primers^26^.

### Definitions

VR in this study was defined as double-negative serum HBV DNA and RNA. SR was defined as HBeAg seroconversion in HBeAg-positive patients. HBeAg seroconversion was defined as the loss of HBeAg accompanied by detection of anti-HBe antibodies.

### Quantitative anti-HBc and HBV RNA evaluation

Serum samples collected at each visit (before treatment, week 24, week 48, week 96, year 5 and year 10) were stored at − 80 °C until analysis. Serum qAnti-HBc was measured using a commercial kit with a double-sandwich immunoassay (WANTAI, China) according to the manufacturer’s protocols and previous studies^11,13,27^. HBV RNA was measured with Diagnostic Kit for Hepatitis B virus pgRNA (PCR-Fluorescence Probing, Lower limit of detection, 300 copies/mL) according to the Manufacturer’s instruction (HOTGEN, China).

### Statistical analysis

The non-normal distributions were showed as median values (Interquartile ranges, IQR), the continuous measurements of normal distribution were represented as means (standard deviations, SDs), and the categorical variables were represented as subject number (percentage). The associations between qAnti-HBc level and VR or SR were assessed using logistic regression analysis. AUROC were calculated for analyses of the performance of qAnti-HBc level in predicting VR and SR. Statistical analysis was performed using SPSS ver. 17.0 software (ISM, Armonk, NY). p < 0.05 was considered statistically significant.

## Supplementary Information

Supplementary Information.
