# Supplementary material for: Baseline quantitative HBcAb strongly predicts undetectable HBV DNA and RNA in chronic hepatitis B patients treated with entecavir for 10 years
Source: Sci Rep. 2021 Jun 28;11:13389. doi: 10.1038/s41598-021-92757-0 (PMC8238999; doi:10.1038/s41598-021-92757-0)
Supplement: Supplementary file 1 — Supplementary Information. [file 41598_2021_92757_MOESM1_ESM.pdf]

Supplementary Information

Baseline quantitative HBcAb strongly predicts undetectable HBV DNA and RNA in chronic hepatitis B patients treated with entecavir for 10 years

Xi Zhang[1#], Xiaocui An[1,2#], Lei Shi[1], Xueliang Yang[1], Yunru Chen[1], Xiaojing Liu[1],

Jianzhou Li[1], Feng Ye[1*], Shumei Lin[1*]

1 Department of Infectious Diseases, First Affiliated Hospital of Xi’an Jiaotong University, Xi’an 710061, China

2 Department of Infectious Diseases, Hospital of traditional Chinese medicine of Yuyang District, Yulin 719000, China

[#] These authors contributed equally to this work and share first authorship.

*Correspondence should be sent to:

Dr. Shumei Lin, Department of Infectious Diseases, First Affiliated Hospital of Xi’an Jiaotong University, No. 277 Yanta West Road, Xi’an City, Shaanxi Province, China; email, linshumei123@126.com; telephone, +86-29-85323262; fax, +86-29-85323722.

Feng Ye, Department of Infectious Diseases, First Affiliated Hospital of Xi’an Jiaotong University, No. 277 Yanta West Road, Xi’an City, Shaanxi Province, China; email, yefeng.jiaotong@163.com; telephone, +86-29-85323262; fax, +86-29-85323722.

Supplementary table 1 Dynamic changes of liver function and virology indexes during entecavir therapy

Characteristics	Baseline	Week 24	Week 48	Week 96	Year 5	Year 10	P value
Serum ALT(IU/L)	104.73±19.82	48.37±5.68	44.89±6.08	38.67±5.13	32.27±4.16	34.35±5.34	<0.001
Serum AST(IU/L)	81.55±11.6	41.52±5.39	34.63±2.16	32.93±2.28	28.42±1.91	26.05±2.02	<0.001
Serum HBV DNA (log10 IU/mL)	6.29±1.21	3.80±0.32	2.80±0.40	1.91±0.40	0.43±0.21	0.55±0.24	<0.001
Serum HBcAb (log10 IU/mL)	3.07±0.87	2.91±0.14	2.80±0.16	2.49±0.17	2.20±0.16	1.93±0.15	<0.001
Serum HBV RNA (log10 copies/mL)	5.39±1.47	4.53±0.31	4.28±0.33	3.59±0.30	3.13±0.26	2.75±1.71	<0.001
